# The Visitor Effect on Zoo Animals: Implications and Opportunities for Zoo Animal Welfare

**DOI:** 10.3390/ani9060366

**Published:** 2019-06-17

**Authors:** Sally L. Sherwen, Paul H. Hemsworth

**Affiliations:** 1 Wildlife Conservation and Science, Zoos Victoria, Victoria, Melbourne 3052, Australia; 2 The Animal Welfare Science Centre, the University of Melbourne, Victoria, Melbourne 3052, Australia; phh@unimelb.edu.au

**Keywords:** zoo animal welfare, animal behaviour, visitor effects, human-animal relationships, stress physiology

## Abstract

**Simple Summary:**

Research has shown that an animal’s welfare is highly dependent on how various individual animal factors (e.g., species traits, genetics, temperament and previous experience) interact with environmental features (e.g., social grouping, enclosure design and sensory environment). One prominent feature of a zoo’s environment is the presence of visitors. Decades of research on the visitor effect in zoos has demonstrated that visitors can have negative, neutral or positive impacts on zoo animal behaviour and welfare. This paper reviews the literature on the implications and potential opportunities of human–zoo animal interactions on animal behaviour and welfare, with the aim of stimulating interest, understanding and exploration of this important subject.

**Abstract:**

Achieving and maintaining high standards of animal welfare is critical to the success of a modern zoo. Research has shown that an animal’s welfare is highly dependent on how various individual animal factors (e.g., species traits, genetics, temperament and previous experience) interact with environmental features (e.g., social grouping, enclosure design and sensory environment). One prominent feature of the zoo environment is the presence of visitors. Visitor contact can be unpredictable and intense, particularly in terms of auditory and visual interaction. Depending on an animal’s perception of this interaction, visitors can have either negative, neutral or positive impacts on zoo animal behaviour and welfare. This paper reviews the literature on the implications and potential opportunities of human-zoo animal interactions on animal behaviour and welfare, with the aim of stimulating interest, understanding and exploration of this important subject. The literature to date presents a mixed range of findings on the topic. It is possible this variation in the responses of zoo animals to visitors may be due to species-specific differences, the nature and intensity of the visitor interactions, enclosure design, and individual animal characteristics. Analysing these studies and better understanding animal preferences and motivations can provide insight into what animals find negatively and positively reinforcing in terms of visitor contact in a specific zoo setting. This understanding can then be applied to either safeguard welfare in cases where visitors can have a negative impact, or, conversely, it can be applied to highlight opportunities to encourage animal-visitor interaction in situations where animals experience positive emotions associated with visitor interaction.

## 1. Introduction

### 1.1. Zoo Animal Welfare

Although personal and community views vary worldwide, it is well recognized that there is a social movement emerging that has led to increased public concern and interest in the welfare of animals in captivity [1,2,3,4,5]. There is a growing public expectation that high standards of welfare in all animal-based industries should be achieved and maintained [6]. Zoos are one of these animal-based industries that have experienced increased public scrutiny and are unique in that the standards of care and welfare of the animals are generally on display to the public and, therefore, open to judgement.

It is likely that the considerable advancements in our scientific understanding of sentience in many species, and their capacity to experience a suite of emotions [7,8], has driven this ethical reflection and raised questions around the quality of life some wild animals experience in zoos and whether their needs can be met to an acceptable standard [7]. As such, this scrutiny may be expected to intensify as our scientific understanding of animal welfare develops further [8].

For zoos to begin to address public concern over animal welfare standards, it is critical that zoos are engaged with the science of animal welfare [9]. This field of science attempts to make inferences about how animals feel based on a number of welfare indicators such as behaviour, endocrine function and physical health [10]. The goal is to provide objective data that can inform advancements in housing and husbandry conditions that facilitate better animal welfare standards. In recent years, the zoo industry has demonstrated significant intensification of zoo-based welfare science, with research being published on a wide range of topics [9,11]. Ward and colleagues [10] summarise the various recent advancements in applied animal welfare science in zoos, highlighting five themes in particular that have experienced expansion including (a) human–animal interactions, (b) anticipatory behaviour, (c) cognitive enrichment, (d) behavioural-biology based husbandry and (e) reproductive and population management. This paper focusses on one of these themes in detail, human-animal interactions, and more specifically, the effect of visitors on zoo animal behaviour and welfare.

Visitors are a prominent feature of the zoo animal’s environment, particularly the frequent, and at times close and intense, interactions. However, the literature on the effects of visitor interactions on zoo animals is somewhat ambiguous, with studies providing evidence for a range of impacts interpreted as negative [12,13], neutral [14,15] and positive [16]. This variation in the responses of zoo animals to visitors may be due to species-specific differences, the nature and intensity of the visitor interactions, differences in the physical features of enclosures, and individual animal characteristics (e.g., past experiences and temperament). Research on this topic has expanded considerably in the past several years. As such, it is time to review the literature on the implications of human–zoo animal interactions on zoo animal behaviour and welfare, with the aim of stimulating further interest, understanding and exploration of this important subject.

### 1.2. The Human Environment in Captive Animal Settings

The study of human impacts on animal welfare in captive settings first arose in the production animal industry in the late 1970s when numerous studies showed wide variation in basic welfare outcomes, even when animals were kept in similar physical environments [17]. At this time, researchers began to recognise the crucial role of the human dimension, including human attitudes and behaviours, in determining animal welfare outcomes in captive settings. Since then, this field of research has developed into its own sub-speciality within the broader field of welfare science and is referred to as Human–Animal Relationship (HAR) research. Most of this research has been conducted in the livestock industry and has demonstrated that HAR is based on the history of interactions between the human and animal, and each individual partner’s experience of the relationship allows it to learn and to anticipate future interactions and as a result, behave accordingly [18]. Thus, this history of interactions leads to the development of learned responses to humans.

The most studied aspect of the HAR in farm animals has focused on interactions that lead to poor welfare outcomes as a result of the animal’s fear response to humans [18]. Research showed that human interactions such as hitting, shouting, rough handling, sudden movements and loud noises can be perceived as negative by the animals and, therefore, increase fear of humans [19]. This fear of humans has been shown to have profound impacts on animal welfare and productivity outcomes. For example, Breuer et al. [20] found that at dairy farms where milk yield was low, cows showed greater fear of humans, as measured by human approach tests. Similarly, in poultry, high fear of humans was associated with reduced egg production, growth and product quality [21]. However, there has been increasing appreciation that animals can also experience pleasant emotions associated with humans that may arise from rewarding events or positive interactions, such as gentle handling and petting [18]. For example, studies have also demonstrated that stroking animals in a similar manner to intraspecific allogrooming reduces heart rate and results in relaxed body postures in cattle [22] and lambs [23]. How an animal perceives human interactions is not only influenced by the nature of the interaction but also by other variables, including previous experience with humans, temperament and motivational state, genetics and species [21,24,25].

The HAR has also been studied in companion animals, with similar patterns detected as found in the livestock industry. One study looked at the impacts of a 45 min human contact session on shelter dogs and found that dogs that were exposed to these human contact sessions had lower glucocorticoid concentration than dogs that were not exposed to this human contact [26]. Similarly, in laboratory studies, the HAR has been demonstrated to impact animal welfare. One study on chimpanzees showed that an additional 10 min a day of positive interaction with a familiar caretaker resulted in chimpanzees grooming each other more and displaying lower levels of abnormal behaviour [27]. Similarly, common marmosets showed increased grooming and playful activities following periods of additional positive interactions with their caretakers [28].

In zoos, the HAR has been much less studied compared to these other settings, but there has been a steady increase in the number of studies recently published on this topic. It is important to acknowledge the key differences in zoos compared to other animal settings. These differences may explain the slower progress in understanding this area of research. Firstly, there is huge variation in the number of species housed at zoos. This introduces a range of challenges in understanding species-specific factors that can influence an animal’s response to humans. Focusing on fear of humans as a key determinant of the direction of the HAR, researchers have suggested that all species have some natural fear of humans and are likely to perceive them as potential predators, at least in their first interaction [29]. It is the level of fear that will vary between species according to certain evolutionary history factors, such as whether they are a prey species or a predator or possess traits such as boldness or shyness [29,30]. Furthermore, zoo species have been described as captive ‘wild’ animals, as they have not gone through the thousands of years of domestication that livestock and companion animals have. Domestic animals have adapted to humans and the captive environment through genetic changes over generations and environmentally-induced developmental events reoccurring for each generation [31]. Zoo animals have no doubt undergone some level of ‘natural selection’ in their captive environment, however, at an industry-level, they have not been purposefully bred for traits associated with adaptation to humans. This creates a different starting point to study the HAR in zoo animals compared to other captive animals. Nonetheless, it is clear that zoo animals are regularly exposed to both familiar and unfamiliar humans in their environment, and, as such, it is possible that this human interaction will have an impact on their welfare.

A model of the HAR for the zoo setting was proposed by Hosey [25], then refined and updated in 2013 [30] and presented by Ward and Sherwen [32]. This model suggests that HARs in zoos can be characterised in three ways: (i) a negative relationship, where the animal is highly fearful of humans and shows avoidance, (ii) a neutral relationship, if interactions with humans have no consequences for the animal and can lead to habituation to humans, or (iii) a positive relationship, where the animal will potentially experience positive emotions associated with the interaction [24,25,30]. Hosey [25,30] developed this model based on Hediger’s [33] early suggestion that humans could potentially be significant to animals in one of five ways; as an enemy, prey, a symbiont, an inanimate part of the environment or as a member of the same species. Each of these scenarios is likely to present different welfare outcomes for the animal. For example, if an animal perceives humans as an enemy (predator or competition), this could lead to fear responses being frequently evoked and, as a result, poor welfare outcomes. However, if humans are perceived as a symbiont, this could potentially be a source of enrichment and produce positive welfare outcomes. Lastly, if animals perceive humans as an inanimate part of the environment, this is likely to lead to a neutral relationship with no consequence for the animal [30].

Zookeepers are the frontline in the delivery of provisions for animals. As such, they have enormous potential to influence animal welfare outcomes in the animals in their care. Keepers feed, clean, train and provide enrichment to the animals in their care, usually on a daily basis, and over time these interactions will develop into a relationship [32]. In comparison to the research that has been conducted in the domestic animal industries, research on keeper–animal interactions in zoos has received very little attention [34], with only a few studies attempting to evaluate this relationship [35,36,37,38,39,40]. For example, Chelluri et al. [36] found that gorillas and chimpanzees showed less self-directed behaviour but more agonistic behaviour in unstructured sessions consisting of one or more positive keeper interactions. Carrasco et al. [37]. found that gorillas who underwent training and play sessions with keepers showed lower rates of abnormal behaviour and higher levels of affiliative and intraspecies play behaviour. Ward and Melfi [38] found differences in the latency of black rhinoceros and Chapman’s zebras to appropriately respond to behavioural cues for different keepers, concluding that unique dyads were formed between keepers and these animals. Other studies have investigated relationships between a keeper’s husbandry style and various biological variables in several populations of animals. For example, a significant positive relationship was found between reproductive success in small felids and a husbandry style in which keepers spent more time talking to and interacting with the cats [39] and lower faecal glucocorticoid metabolite concentrations in clouded leopards were found to be associated with two key husbandry variables, a greater amount of time keepers spent with the leopards and a lower number of keepers caring for the animals [40] For more detailed reviews on this topic, see Hemsworth et al. [18] and Ward and Sherwen [32]. The target of this review paper is the visitor effect on zoo animals, which has considerably more research.

### 1.3. Zoo Visitors

The presence of visitors is a universal characteristic of the zoo environment. It is in the best interests of zoos to attract visitors and provide a good visitor experience. This is because visitors are key to delivering many zoo-based conservation goals as the targets of many social conservation education and advocacy campaigns designed to mitigate human-driven threats to wildlife [41]. Furthermore, visitors can also contribute financially to zoo-based conservation through donations and entry fees. Research has shown visitors care about animal welfare [42], and any signs of suffering or welfare compromise in zoo-housed animals can counter-balance a zoo’s contribution to conservation. Conversely, a positive visitor experience as a result of viewing animals engaged in natural, active behaviours has been shown to facilitate learning in zoos [41,43]. Ultimately, the more community support a zoo gains, the better placed the zoo will be to deliver both conservation and animal welfare goals.

This makes visitor experience and animal welfare inextricably linked and highlights the importance of research in this area. Clearly the goal is for zoos to provide high standards of welfare, ensure animals are living well and showcase this to visitors. If an animal displays signs of stress in the presence of visitors or shows avoidance, there is potential for a conflict between these goals. Alternatively, for some species and individuals, it is possible that visitors can provide a source of stimulation or contribute to other positive experiences for animals, providing opportunity for enhanced animal welfare and visitor experience. Thus, research into understanding this visitor-animal relationship is critical to inform the management of these potential conflicts or opportunities.

Hosey [44] described the term ‘visitor effect’ in reference to the study on the impacts of zoo visitors on animals, with systematic investigations into this topic largely beginning in the late 1980s and 1990s and growing over time. As Hosey’s [25] model predicts, the visitor effect studies conducted to date have provided evidence for negative, neutral and positive relationships. The various studies will be discussed below, but first it is useful to highlight the methodology that has typically been used to study the impact of visitors on zoo animals to date.

## 2. Assessment of the Visitor Effect

### 2.1. Metrics Studied

There have been considerable advances in animal welfare science that have led to the development of a range of validated measures of animal welfare. The majority of measures available have focused on identifying indicators of negative welfare. However, as the science of animal welfare has advanced, it is now well recognised that providing for animal welfare should not only include minimising suffering but also the provision of conditions that result in satiety and contentment [45,46,47]. The validation of indicators of positive welfare is an emerging area of investigation in welfare science [45]. Reflecting this historical focus, most visitor effect studies have targeted indicators of negative welfare in animals.

By far the most common approach to study visitor effects in zoos has been to assess behavioural changes in animals in response to different visitor conditions. Behaviours such as aggression, avoidance and stereotypies have been used as indicators of negative welfare states, particularly associated with fear and stress [30,48,49]. In contrast, exploratory, play and affiliative behaviours have been used as indicators of positive welfare states [45,47].

However, it is important to be cautious with interpretation of some of these behaviours. A large proportion of an animal’s behavioural repertoire is likely to consist of behaviours that have no clear implications for welfare, such as locomotion, vigilance and resting. For these behaviours, it may be useful to analyse any changes in time spent engaged in them in response to any changes in visitor conditions as a measure of how visitors can affect the behaviour of the animal, but welfare consequences cannot be inferred from these changes alone. For example, increases in vigilance behaviour in zoo animals in response to increasing visitor numbers could reflect interest in visitors, and may therefore be considered stimulating for animals. Alternatively, it could be an anti-predator behaviour, which may result in fear and anxiety and consequently stress. To help assess whether changes in such behaviours have adverse implications for welfare, physiological stress should also be monitored. Methods to monitor this are discussed further below.

Behavioural measures that are commonly considered indicators of stress and poor welfare include aggression, avoidance and stereotypies. For example, aggression can compromise welfare if it results in injury [50] and has also been widely associated with physiological stress [47,51]. Avoidance of humans as a measure of fear of humans has been validated on the basis of findings of behavioural and physiological correlates of fear in animals [18] and avoidance behaviours have been widely used as an indicator of fear of humans in zoo animals [52,53]. Stereotypies in zoo animals are the most studied behavioural indicator of poor welfare, perhaps because they tend to receive a lot of attention particularly in zoos as they can be relatively well recognized by the public [42]. The nature of the expression of stereotypic behaviour is diverse across species and individuals. However, some common examples include self-scratching or self-mutilatory behaviour in primates [54,55,56], pacing in carnivores [57] and oral stereotypies, such as non-food object licking in ungulates [58]. Although there is a considerable amount of evidence that suggests the expression of stereotypies is linked with compromised welfare, there are instances where there is a poor relationship between stereotypies and stress, particularly in situations in which frustration-induced stress may be at least partly resolved if the behaviour expressed reduces the underlying motivation [59]. For example, in farmed mink, stereotypy performance has been shown to be negatively correlated with glucocorticoid (GC) concentrations (see the review in Mason and Latham [60]). This, again, highlights the importance of being cautious with interpretations of just one measure of welfare when investigating visitor effects and other environmental effects.

The expression of many of these behaviours can certainly indicate that the environment is sub-optimal, and visitors are having an impact on the animal’s behavior. However, to analyse whether the effect has physiological consequences for the animal, more direct measures of physiological stress and fitness consequences are required [61,62]. Similar to behavioural indicators of stress, research including physiological indicators of stress has also been focused towards identifying indicators of negative welfare. As a result, we have a much better understanding of how we can measure the stress response in animals, but currently lack well established physiological indicators of positive welfare in animals. However, there are some promising developments in this area around the potential use of salivary IgA and heart rate variability, particularly the cardiac vagal tone [45]. However, such methods have not yet been widely applied to zoo settings.

Measuring the concentration of GC can provide insight into stress physiology, and hence the welfare of animals in relation to various environmental conditions, including visitor exposure [63,64]. Plasma GC concentrations are widely used to assess stress responses in various species. However, there are obvious constraints of the blood sampling procedure for zoos, as it involves the capture and handling of animals to collect samples. This is not only logistically difficult (and dangerous) for many zoo species, but it is also invasive, and circulating GC concentrations can be affected rapidly (within minutes) in response to the stress of handling and restraint, which can substantially alter the physiological parameters that are under investigation [65,66]. Furthermore, there is marked variation in plasma GC concentrations at any point in time because of pulsatile secretion patterns and circadian rhythms [67].

More recently, less invasive techniques have been developed that enable the analysis of stress hormone metabolite concentrations in matrices such as saliva, urine, faeces and even hair, feathers and eggs [68]. Non-invasive sampling techniques can overcome some of these above-mentioned problems with plasma sampling. In zoo settings, the most common way to measure GCs has been through faecal sampling. This technique offers the advantage of representing the average GC metabolite concentration output over hours or days [69] and, therefore, represents pooled quantities of GC secretion from the adrenal medulla (i.e., baseline plasma GC), dampening pulsatile effects [67,70].

### 2.2. Study Design Approaches

There have been, to date, four main approaches to studying the visitor effect in zoos. The most common of these approaches has been to focus on site-specific data and analyse relationships between animal behaviour and natural variation in visitor conditions. These are correlation studies. This type of study can make interpretation of visitor effects difficult because of the influence of confounding variables that can also be associated with variation in visitor numbers, such as weather conditions, time of year and possible changes in husbandry routine [71]. Goodenough and colleagues [72] suggest that visitor effects may have been overestimated for this very reason. They studied the effect of visitors and other environmental variables, such as weather, on ring-tailed lemur behaviour and found that time of day and weather exerted the strongest effect on lemur behaviour. Their results suggested visitor variables accounted for around 20% of the behavioural variation observed in lemurs, but this dropped to around 6% after time of day and weather were included as covariates in the model [72]. The authors recommend that these associated environmental variables are carefully considered in future studies.

It is also worthy to note that it may actually be the case that animal behaviour is influencing visitor numbers at enclosures, rather than visitors influencing animals [15,44,73]. This is based on the notion that zoo visitors are likely to be more attracted to animals that are engaged in active behaviours. There is certainly some evidence to support this thinking. For example, Bitgood et al. [74] found that across all taxa at all study zoos, visitors spent more time watching animals when they were active than when they were inactive. Similarly, Margulis [15] found that visitor interest increased when felids were more active. Although many of these correlational studies do not demonstrate causality, they do provide a rationale for further experimental research in which visitor variables are manipulated in a controlled manner.

A handful of studies have used this approach to study visitor effects. Chamove et al. [75] asked visitors to either stand or crouch at the viewing window for cotton-top tamarins, Diana monkeys and ring-tailed lemurs and found there was less agonistic behaviour and more grooming behaviour when the visitors were crouching rather than standing. Several studies have investigated the effect of visual barriers that obscure or block the view of visitors on various species of primates including gorillas [12,61], orangutans [16] and black capped capuchins [52]. The visual barrier treatments resulted in significantly less aggressive behavior and abnormal behavior in both the gorilla study [12] and the black capped capuchin study [52]. The orangutan study [16] was designed as more of a preference test with half of the window covered with a visual barrier and the other half left open. It was found that the orangutans showed a preference to position themselves in front of the open window. Other studies have experimentally blocked access to visitors on some days to investigate the effect of presence or absence of visitors on animals [53,76]. When visitors were blocked from an exhibit, little penguins showed less aggression, huddling and avoidance behavior [53], and when visitors were blocked from a quokka exhibit, more quokkas were visible from the visitor pathways [76]. Mitchell et al. [73] moved golden bellied mangabeys between enclosures that varied in exposure to visitor numbers and found that transfer to enclosures where the mangabeys were exposed to more visitors increased their aggressive displays towards visitors and intra-group aggression. Others have manipulated visitor noise levels by asking groups to be quiet in the vicinity of target animal enclosures [14,77] or by exposing animals to crowd noise audio recordings and analysing behavioural responses [78]. For example, exposure of koalas to ‘loud’ crowd noise playbacks increased their behaviours indicative of disturbance compared with ‘quiet’ crowd noise playbacks [78]. These experimental studies are necessary to determine the causality of visitor contact on animal behaviour and welfare. This knowledge can be of significant value to zoos in informing management decisions.

Multi-species comparative studies are another approach that has been used to study visitor effects. This approach involves the study of visitor effects on a range of different species, most commonly from the same taxonomic group [15,75,79]. For example, Chamove and Hosey [75] investigated the relationships between the body size of 12 species of primates and their responses to visitors, based on the hypothesis that small species may be particularly prone to effects from visitors. They found a negative correlation between body size and the level of activity in the primates. However, the trend was not statistically significant. Another study investigated a range of species across different taxonomic groups [80], to understand if there are species-specific traits, such as body size, diet or activity cycle associated with an animal’s response to visitors. The main aim of this approach is to understand if these species-level factors could explain an animal’s response to visitors, and, as a result, allow zoos to predict which species are likely to be most and least impacted by visitors. The authors found significant differences in locomotor and resting behaviour in different species in response to different visitor categories of small, medium and large crowd sizes and noise levels. The factors that most explained responses to visitor categories were habitat and activity cycle, with the species from closed habitats compared to those from open habitats, and diurnal species compared to nocturnal species showing more behavioural changes in response to visitors [80]. These species-level factors are discussed further below.

The last of the four main approaches to studying visitor effects involves multi-institutional comparisons that aim to investigate relationships between animal measures, such as faecal GC metabolite concentration or display of abnormal behaviour and a range of environmental variables. These studies typically are not designed to focus on visitor effects, but they have highlighted significant relationships between animal welfare measures and exposure to visitors. For example, Carlstead and Brown [70] found that black rhinos had higher mean faecal GC metabolite concentrations at zoos where rhinos were housed in enclosures that were exposed to the public around a greater proportion of the perimeter. Pirovino et al. [81] found that pileated gibbons with more visual protection from visitors had lower average faecal GC metabolite concentrations. Similarly, Wielebnowski et al. [40] focused on faecal GC metabolite concentration but studied clouded leopards and found that animals on public display had higher average GC concentrations than animals off display. These multi-institutional studies are particularly useful in identifying environmental features that allow animals to behaviourally regulate their interactions with visitors.

## 3. Direction of the Effect

This section aims to highlight the direction of the effects that have been reported. As discussed above, there is evidence that visitors can have a range of effects on animals. These effects have been interpreted as positive, neutral and negative. Table A1 presents a summary of the majority of current studies conducted on visitor effects in zoos. This is not an exhaustive list of studies but covers the main literature highlighting the target of each study and the main results. This table supports the following synopsis of the literature on visitor effects.

### 3.1. Negative Impacts

The majority of studies on this topic have concluded that the effects of visitors on animal behaviour and welfare can be interpreted as negative. It should be noted that it is possible that this publication bias is a result of the traditional focus of welfare science on identifying and managing negative welfare states in animals, resulting in increased attention to these concerns for captive animals and our advanced ability to identify them as a result of a suite of well-recognised indicators of poor animal welfare.

Negative responses in zoo animals to humans are likely driven by fear. In the wild, fear plays a crucial role in escaping predators by motivating animals to avoid potentially harmful situations [82,83]. As mentioned above, fear can be triggered by environmental stimuli that are novel and have high intensity, such as loud noises, large size or sudden movement [83]. If zoo visitors behave in a way that is loud, fast and unexpected in the presence of animals, these actions may be threatening for some zoo species. Behaviours such as avoidance (fleeing or retreating), inhibition of movement (freezing), aggression, vigilance and certain vocalisations in the presence of humans have been assessed in many domestic animal studies as indicators of fear of humans [18]. In zoos, similar measures have been used to study the impact of visitors and make inferences about the likely welfare impact.

Evidence for avoidance of visitors has been found in several studies. The presence of visitors was associated with fewer animals being visible in a group of quokkas [76] and an increase in hiding behaviour and distance from the visitor viewing area in little penguins [53]. Moreover, increasing visitor numbers were associated with less time visible to the public in a range of species, including orangutans [77], jaguars [84] and siamangs [85]. An increase in the time spent alert to visitors was detected in various species including gorillas [61], kangaroos [86], sika deer [87], koalas [78] and Soemmerring’s gazelle [88] in response to higher numbers of visitors. Higher rates of aggression associated with visitor presence were noted in baboons [89], Indian gaur [90] and cotton-top tamarins [91] and greater visitor numbers were associated with higher rates of aggression in mangabeys [73], mandrills [75] and gorillas [92]. An increase in time spent engaged in stereotypic behaviour with increased visitor numbers was observed in gorillas [54,93] pileated gibbon [94], jaguar [84], fennec foxes [35] and brown bears [95].

Another behavioural change that has been noted in some species in response to different visitor conditions is a deviation in activity budget. These behavioural changes are more difficult to evaluate for welfare implications, as discussed above, but are nonetheless interesting to note, as they may potentially result in poorer welfare outcomes if certain behaviours are restricted as a result of visitor presence. For example, higher visitor numbers have been associated with lower frequencies of foraging, grooming and play in chimpanzees [96], less time spent lying and eating in Mexican wolves [97], more time inactive in pumas [98] and less time spent swimming in little penguins [53] and African penguins [99]. As discussed earlier, studies that investigated physiological changes associated with visitor conditions provide another insight into potential welfare concerns, and several of these studies exist in the visitor effect literature. An increase in visitor numbers was associated with increased urinary GC concentrations in spider monkeys [13] and faecal GC concentrations in Mexican wolves [97] and blackbuck [100]. The presence of a one-way viewing screen that reduced the view of visitors resulted in a reduction in faecal GC concentrations in black-capped capuchins [52].

Situations in which visitors have negative impacts on animals can be a concern for zoos because of the risks to both animal welfare and visitor experience. If visitors are perceived by zoo animals as fear provoking or stressful stimuli, long term exposure to visitors could be a source of repetitive acute or chronic stress. Additionally, many of the changes in animal behaviours mentioned above such as avoidance, hiding, aggression and reduction in play and activity are likely to have implications for visitor experience. It is, therefore critical for zoos to fully understand these relationships so that risks of negative visitor effects can be mitigated or managed.

### 3.2. Neutral Effects

Several studies suggest that visitors have no impact on animal behaviour or welfare. For example, a study on meerkats investigated the effect of reducing the intensity of visitor behaviour (e.g., noise levels and attempted interaction) on several groups of meerkats and found no difference in meerkat behaviour at any site in response to the experimental reduction in intensity of visitor behaviour [14]. Similarly, O’Donovan [101] found that visitor numbers and noise level had no effect on cheetahs and Margulis and colleagues [15] found no effect of visitor presence on several species of large felids.

It is possible that a lack of response to visitors (a neutral response) may be a result of zoo animals becoming habituated to visitors, resulting in perceptions of visitors as an inanimate or a non-threatening part of their environment. It has been well documented that individuals of some species that have benign interactions with humans in the wild undergo habituation that can lead to a degree of human-tolerance [29]. This has been reported in several wild populations of various species including Magellanic penguins [102], Gunther’s dik-diks [103], gorillas [104] and brown bears [105]. Therefore, habituation to zoo visitors is likely to occur in zoo animals if repeated exposure to visitors has neither rewarding nor punishing elements and is, therefore, of no consequence for the animal [32].

Habituation to visitors is an important factor to consider in visitor effect studies and, for some zoos, a situation in which animals ignore visitors and go about their daily activities regardless of crowds, might be the ultimate goal, if the focus is on natural behaviours. Alternatively, some zoos might want to encourage interaction between visitors and animals, with the aim of improving visitor experience. In these cases, it may be important for the visitor effects to be predominantly positive rather than neutral.

Lastly, it remains possible that the smaller number of studies demonstrating neutral effects of visitors compared to the number of studies demonstrating negative effects is not representative of the situation in zoos. Because of the publication bias mentioned above, research may be more likely to be conducted and published in response to a perceived welfare concern. Thus, it is possible that a neutral response to visitors is far more widespread than the current literature implies.

### 3.3. Positive Effects

In addition to the above-mentioned studies that have provided evidence for both negative and neutral effects of visitors, there is also some limited evidence that suggests visitors can be a positive source of stimulation. One of the few experiments suggesting this kind of relationship studied orangutan location and orientation in relation to several visitor viewing conditions [16]. Three treatments were imposed on the viewing window including: window uncovered, left side of window covered and right side of window covered. It was found that manipulation of visitor viewing conditions resulted in the orangutans preferring to position themselves to face the window of the visitor viewing area. Since there was no evidence of avoidance of visual contact with visitors, one interpretation of these results is that the orangutans were attracted to viewing the visitors themselves, rather than the visitor viewing area. Clearly this is an interesting finding that requires further investigation.

There are also several cases of animals working to initiate interaction with visitors. For example, a corella was reported to spend more time engaged in ‘attention-seeking’ behaviours to initiate interaction with visitors when fewer visitors were present [106], and chimpanzees at Chester Zoo initiated interaction with visitors, particularly if soliciting food [107]. Prairie dogs were found to move closer to visitors under higher visitor numbers [108]. Diana monkeys increased the time spent playing and feeding when greater numbers of visitors were present [109]. Given that play behaviour is considered an indicator of positive animal welfare [45], it is possible that this group of monkeys was also positively stimulated by visitors. Another study investigated the effect of visitor numbers on behavioural diversity and pool use in Gentoo penguins [110]. The authors found that higher numbers of visitors were associated with greater behavioural diversity and increased pool use by penguins. These results indicate that these Gentoo penguins were not negatively affected by visitors, but instead were more active, which the authors interpret as a positive response, since penguins are pelagic birds that naturally spend large amounts of time foraging at sea, contributing to a higher overall level of behavioural diversity.

There are many anecdotal reports from zoo professionals that suggest some zoo animals solicit interaction from visitors at times. Although empirical evidence for positive effects of visitors on zoo animals is sparse, clearly the topic of whether visitors or properties of visitors are positively reinforcing for some species in some zoo settings requires rigorous investigation because of its potential value to both the animal and visitors. Preference and motivation testing can be applied in captive settings to tell us what animals find negatively and positively reinforcing [111]. This understanding of what features of visitor contact can potentially be positively reinforcing provides opportunities for zoo animals to experience positive emotions associated with visitor interaction. As highlighted by Dawkins [111], it is important to conduct these studies in situ so the results are directly applicable to the environment in which the animal lives. One study on Galapagos giant tortoises in a zoo examined individual preferences in enrichment type, comparing objects (e.g., balls) with a keeper scrubbing the shells and rubbing the necks of the tortoises. All individuals showed a preference for keeper interaction over object enrichment [112]. Preference and motivation research provides the opportunity to identify the preferred environmental resources and behavioural opportunities important to the animal.

## 4. Discussion of Possible Explanatory Factors

Clearly the effects of visitor interactions on zoo animals are inconsistent. Some interactions may be stressful, innocuous or possibly enriching for animals. It is useful to consider the factors that may influence an animal’s response to visitors, such as species evolutionary traits, individual animal traits and environmental features of the enclosure. These factors may affect how the animal responds either individually or in combination with each other.

### 4.1. Species Evolutionary Traits

Research has demonstrated that species vary considerably in their responses to captivity, even among close taxonomic relatives (for a review, see Mason [113]). Many species breed successfully and have longevity records greater than their wild counterparts, whereas others fare worse than expected with poorer longevity in zoos than in the wild and show susceptibility to ‘stress related illnesses’ [113]. Broom [114] suggests that the evolution of animals in their natural environment has resulted in each species having certain tolerable physiological limits to allow coping, as well as certain behavioural or psychological needs. Researchers have suggested that an animal may experience suffering if they are unable to adequately perform relevant activities [115].

These species traits are highly likely to influence an animal’s response to visitors. The challenge is that of the published studies on the visitor effect to date, it is difficult to draw any conclusions about the extent to which species traits impact an animal’s response to visitors because of the disproportionate representation of studies across taxa, with most studies on non-human primate species (Table A1, [30,116]), as well as the large variation in the methodology used to assess animal welfare. Nevertheless, limited evidence does exist that suggests several species traits associated with their life history characteristics are likely to influence an animal’s response to visitors, as first highlighted by Hediger [33].

For example, researchers have highlighted variation in fear of humans across species [25,117,118]. Young baboons were found to be more fearful of humans than young Rhesus macaques [119]. This is likely dependent on an animal’s life history, with the response of a prey species to humans likely to differ from the response of a large predatory species [30]. To examine this further, it is useful to consider the concept of predator naivety that is often pronounced on islands where species are found with few or no predators [120]. Macropods from islands compared with macropods on mainland Australia are a good model to consider. One visitor effect study detected subtle differences in the response to visitors in two species of kangaroo, one from mainland Australia (the red kangaroo) and one from a predator-free island (Kangaroo-Island kangaroo) housed in the same zoo enclosure [86]. The study found that red kangaroos (the species exposed to natural predation) spent a considerable proportion of their time engaged in visitor-directed vigilance (15–50%) compared to Kangaroo Island kangaroos (less than 2%) and consistently positioned themselves an average of 4 m further away from the visitor pathway compared to the island kangaroos. It is possible that these behavioural differences exist because the island kangaroos are naturally less fearful of humans compared to the red kangaroos that have experienced a strong evolutionary history of predation pressure. Such factors are likely to predispose species tendency to develop fearful responses to humans in zoos and clearly requires further experimentation.

Margulis and colleagues [15] have also suggested that primates are more responsive to outside-enclosure disturbances in zoos in comparison to big cat species, and that this might reflect the different communication modes between the groups. The close relatedness to humans may also play a role in primate response to visitors as some facial expressions and visual gestures are homologous with human gestures [121,122]. This may expose primates to increased signals of aggression, particularly if visual gestures are perceived as threatening [122], such as direct eye contact [123]. In support of this notion, there are several studies on the aggressive behaviour of nonhuman primates directed towards visitors. For mandrills [75] and golden-bellied mangabeys, threats towards visitors increased with high visitor numbers [73]. Siamangs were more hostile when visitors mimicked hostile siamang behaviour such as staring or yawning [106] and a male orangutan in Birke’s [77] study also displayed increased aggressive behaviours in response to human stares. Sherwen et al. [52] also found that reducing visual contact with visitors significantly decreased both intraspecific aggression and aggression directed towards visitors in black-capped capuchins.

Overall, aside from the studies mentioned above, very little work has been done on identifying species traits that may make them more or less susceptible to negative effects from visitors. Clearly, more research is needed in this area and multi-institutional comparative studies with large sample sizes that investigate correlations between a range of species’ life history variables and visitor effects would be useful to further address this question.

### 4.2. Individual Traits

It is also important to note the contribution of individual differences within a species. An animal’s response to humans will not only be influenced by life history characteristics of a species, but also by individual factors such as genetics (artificial and natural selection), temperament and past experience with humans [31].

Bashaw [124] highlights that an animal’s welfare state is influenced by its perception of the environment in which it lives. This individual perception is, in turn, influenced by the animal’s evolutionary history (as discussed above), temperament and previous experience. As such, two individuals of the same species housed in the same environment and provided with the same husbandry will not necessarily perceive the environment in the same way and, as a result, may have very different welfare outcomes [124,125,126]. Over the past 20 years there has been an exponential increase in the number of studies published on animal temperament and its implications for animal welfare and management in captive institutions [127]. Researchers have highlighted the influence temperament can have on many core zoo goals, including conservation output from captive breeding and reintroduction programs [128,129], as well as zoo population management and social group cohesion [130]. Given the expansion of studies on this topic, it is surprising that little attention has been directed towards understanding the effect of temperament traits on an animal’s response to visitors. Stoinski and colleagues [131] have provided the most thorough investigation into this question to date. They studied four groups of gorillas in one zoo, examining how a range of animal variables (including personality, sex and rearing history) influenced their response to different crowd sizes. Although not statistically significant, the researchers found sex differences in behavior, where males showed a trend towards higher rates of aggression when larger crowds were present. They studied four personality factors, including extroversion, dominance, fearful and understanding, and found a trend towards variation in response to crowd size as a function of the individual personality ratings of these factors.

Some authors have suggested that temperament traits can be heritable and linked to fitness, making them subject to selection pressure in captivity [129]. In captive populations, natural selection is expressed through differential mortality and reproductive failure [31], with an inability to adapt to the close proximity of humans likely to be one of many selection pressures facing captive animals. In addition to heritable temperament traits, an animal’s response to humans is also likely to be heavily influenced by experiential factors, particularly early in life. Studies in the livestock industry have demonstrated that positive conditioning to humans early in life can subsequently reduce an animal’s fear of humans [132,133,134]. However, it is critical to balance this with other species-appropriate early life experiences required for the individual, as various studies have suggested human-only rearing of zoo animals is associated with abnormal behaviour [81] and reproductive problems [135]. For example, hand-reared parrots were found to be more likely to suffer from abnormal repetitive behaviours and were more averse to interacting with humans and enrichment compared to naturally-reared birds [136]. Similar relationships have been demonstrated in a variety of mammalian species. These studies suggest that hand-rearing can result in heightened fear, aggression and stress responses, as well as poor social and parenting skills [135,137]. These studies demonstrate the importance of considering factors that influence an animal’s experience in early, developmental stages of life to ensure there are no undesirable life-long changes in behaviour and impacts on welfare. Clearly, a balance needs to be struck between natural rearing and species-appropriate socialisation and bouts of positive handling of animals by humans early in life to subsequently reduce their fear of humans and thus assist animals in adapting to their captive environments.

### 4.3. Environmental Features

An animal’s physical environment will also influence their response to visitors. Enclosure design, such as the type of barrier between visitors and animals (e.g., wire mesh or glass), size and features in the enclosure to allow animals to approach or avoid visitors, as well as the height and proximity of visitor viewing areas, will determine the animals’ sensory exposure to visitors. A recurring finding across many species is that the ability for an animal to control its exposure to such stimuli can play a major role in how it copes in captivity [138]. A suite of research has demonstrated the positive impacts of giving choice and control to animals, with some studies highlighting that actually having the option of choice can be more important than using it. For example, Ross [139] documented that polar bears showed a reduction in pacing and increased positive social play when given free access to their off-display areas, even though they rarely chose to enter this area. Similarly, Owen [140] found that pandas had lower cortisol levels and fewer signs of behavioural agitation when given the choice of where to spend their time.

With regard to managing visitor effects, it is clear that the ability for an animal to retreat from visitors can reduce or mitigate any stress associated with visitors [116,131,138,141,142]. Thus, enclosure design is of critical importance in allowing animals to control their flight distance from visitors if required. One study directly investigated the impact of access to retreat areas in a petting zoo. Anderson and colleagues [143] manipulated the amount of retreat space available to African pygmy goats and Romanov sheep and found that aggression and escape attempts were lowest when they had access to the full retreat. This concept is supported by studies that have investigated relationships between physiological indicators of welfare and enclosure features. For example, Wielebnowski et al. [40] found a negative correlation between enclosure height and faecal GC concentrations in clouded leopards and interpreted enclosure height as a measure of their ability to avoid contact with other animals and humans. Similarly, a study on 45 individual Canada lynxes across 22 zoos found that lower GC concentrations were associated with a larger enclosure size and a greater number of hiding locations, again highlighting the importance of adequate retreat areas for zoo animals [64]. Carlstead and Brown [70] found that black rhinos had higher mean GC concentrations at zoos when they were maintained in enclosures that had a greater portion of the perimeter exposed to visitors. Further, in black rhinos, breeding success was found to be positively correlated with enclosure size [144]. Moreover, appropriate hiding places and increased enclosure complexity may allow ample space for appropriate escape responses or hiding when animals are confronted with a fear-eliciting stressor.

The design of the enclosure and various features in the environment can also influence visitor behaviour, which, in turn, can have positive or negative consequence for animals. For example, Blaney and Wells [12] introduced a camouflage net to the visitor viewing window to evaluate impacts of obscured visitor visual contact on gorilla behaviour. They also studied the impacts of this enclosure addition on visitor perception and anecdotally reported changes in visitor behaviour as a result of the introduction of this barrier. The authors found that visitor perception of gorillas and their exhibit was more positive when the net was in place and interestingly, they also suggested that visitor behaviour varied markedly between the two conditions with the camouflage net encouraging quieter, more relaxed visitor behaviours, including less time banging on the glass. They also reported that visitors tended to speak less and more quietly when the net was in place. It appears there are various elements of enclosure design that can influence the visitor-animal relationship both directly through ensuring adequate behavioural provisions to allow animals to control their exposure to visitors, as well as indirectly through subtle enclosure features that can affect visitor behaviour at the exhibit, which can, in turn, influence the animal.

## 5. Future Directions

This review has discussed the effects of visitors on zoo animals housed in typical display enclosures but has not discussed the effect of visitors on animals involved in close encounter experiences. Typically, these zoo experiences provide visitors with the opportunity, under the supervision of zoo staff, to photograph and, in some cases, handle or feed the animals. An emerging trend in the zoo industry is the increasing use of these forms of encounters with the assumption that they facilitate a connection between animals and visitors and, therefore, may foster more positive conservation attitudes and behaviours [32,145]. However, very little research has been conducted on this topic from both the animal’s perspective and the visitor’s perspective [32,146].

Research so far has been limited to reports on the behaviour and welfare of dolphins involved in encounters [147,148], felids in behind the scenes experiences [149], crowned lemurs [146] and giraffes [150] in visitor feeding programs, as well as one study on armadillos, red-tailed hawks and hedgehogs involved in an education program [151]. The results of these studies are mixed. One study on dolphins involved in a ‘swim with a dolphin’ program found that refuge use increased in dolphins during and within 15 min following a session [147], whereas another study found an increase in play behaviour in dolphins following an interactive swimming program with visitors when these programs were conducted once per day [148]. A study on giraffes found that time spent engaged in visitor feeding programs had no effect on the performance of stereotypic behaviour [150]. However, Baird and colleagues [151] found that a large amount of handling associated with education programs increased pacing behaviour in armadillos and increased faecal GC concentration in armadillos, hedgehogs and hawks. Given the widespread nature of these experiences offered across zoos, this should be a priority area for research. Similarly, the housing of animals in free range enclosures also appears to be an emerging trend that currently lacks sufficient scientific evaluation. These enclosures are characterised by the lack of physical barriers between visitors and animals, and as a result, the potential for intense and close visitor interaction [86,146]. Currently, only a handful of studies exist that have studied welfare outcomes for animals in these settings [76,86]. Both these studies found behavioural differences in response to different visitor conditions, with increasing visitor numbers, resulting in increased time spent vigilant towards visitors in red kangaroos and Kangaroo Island kangaroos [86], and the presence of visitors resulted in fewer quokkas visible in their enclosure [76]. Large, mixed species bird aviaries are another housing type typically characterised by free-ranging animals and a lack of physical barriers between people and animals. Such enclosures have also been overlooked in the scientific literature. Another aspect that may be an increasing trend associated with these housing types with limited barriers is the increased opportunity for animals to be frequently fed by visitors throughout the day (either by unsanctioned or approved feeding). Depending on the circumstances, uncontrolled visitor feeding may impact animal health and behaviour. For example, visitors feeding animals may result in zoo animals associating visitors with a positive experience, but it may also result in mismanagement of nutrition for certain animals, resulting in health problems. This remains an unstudied area and should be a focus for future research. With the proliferation of these housing types, it is critical for zoos to develop an understanding of any welfare implications or benefits associated with these environments.

Also worthy of consideration is the effect of these more intense, close interactions with animals on visitor experience and attitudes. There have been few targeted, systematic studies on this topic, and, as a result, the impact of participation in these encounter experiences on visitor perception and conservation attitudes remains largely unknown. Emerging research on a related topic has suggested that the presentation of animals in anthropogenic environments has been shown to have a significant impact on public understanding and attitude towards the species’ conservation status. Ross and colleagues [152] found that people who were shown images of chimpanzees in the presence of humans were more likely to believe that wild populations of chimps are stable and would consider owning one as a pet, compared to people who were shown images of chimps without a human present. A recent follow up study further supported this finding and demonstrated that viewers shown images of capuchin monkeys, squirrel monkeys and ring-tailed lemurs in close contact with humans also showed an increased desire to keep these primates as pets, compared to viewers shown images of these same species in natural forested areas [153]. Interestingly, viewers were more likely to describe the animals as appearing “happy” when shown in the absence of people. These studies highlight the potential implications of the way in which animals are depicted and the risk of fostering an uninformed public understanding that can undermine a zoo’s conservation mission.

Finally, it is important to continue to expand the number and diversity of species studied with regard to visitor effects. There is clearly a strong bias towards nonhuman primates in the literature with almost half of all studies on the topic targeting primates (Table A1). However, to the author’s knowledge, there are no studies published for example on the impact of visitors on reptiles, amphibians or fish, despite many species belonging to these taxa being housed in zoos in large numbers. Additionally, the focus has clearly been on studying species that are expected to respond negatively to visitors, but it is becoming equally as pertinent to understand opportunities for positive human–animal relationships in zoos [32] as some species may have positive experiences with visitors, highlighting opportunities to encourage this interaction as part of a human enrichment program for zoo animals. Indicators of this relationship might include play behaviour or evidence of attraction to visitors as revealed by Bloomfield et al. [16].

More studies conducted on a range of species and enclosures are likely to indicate emerging patterns that can assist in more clearly identifying animal and enclosure characteristics that facilitate either negative or positive human–animal interactions and allow zoos to manage accordingly. This information will provide zoos with an evidence-based foundation to inform decision making on species selection in zoos, housing and husbandry approaches and better manage visitor engagement opportunities that will ultimately contribute to conservation success.

## Appendix A

**Table A1:** A summary of the majority of studies published in peer-reviewed journals conducted on the visitor effect highlighting the visitor variables studied, the animal measures used and the main results. * represents studies that involved some form of experimental manipulation. GC, glucocorticoid.

Species	Visitor Variable	Animal Measure	Results	Reference
**Primates**				
Baboon	Presence vs. absence	Behaviour	When transferred to a new enclosure that was on display to the public, the male baboon increased throwing behaviour, which included throwing objects or faeces at visitors or staff.	[89]
Black-capped capuchin	Visual contact *	Behaviour and GCs	Reducing visual contact with visitors resulted in a reduction in group aggression, GC concentration, abnormal behaviour and avoidance of viewing area.	[52]
Chimpanzee	Number	Behaviour	High visitor numbers were associated with lower frequencies of foraging, grooming and play.	[96]
	Interaction sequences	Behaviour	Both chimpanzees and visitors regularly initiated interaction. Chimpanzees interacted with humans primarily to obtain food.	[107]
	Number	Behaviour	There was no effect of crowd size on chimpanzees’ use of the areas of their exhibit closest to zoo visitors. In addition, they were observed in this area at a rate equal to or greater than expected by random movements at all three levels of crowd size analyzed.	[154]
	Number	Behaviour (birth timing)	An analysis of the timing of 231 live chimpanzee births in accredited North American zoos found no weekend (high visitor numbers) or weekday (low visitor numbers) effect on number of births.	[155]
Colombian spider monkey	Number	Urinary cortisol	An increase in visitor numbers was associated with an increase in urinary cortisol concentration.	[13]
Cotton top tamarin	On display vs. off display	Behaviour	The animals on display to the public exhibited less social behaviour than those not displayed to the public.	[91]
Diana monkey	Number	Behaviour	Higher visitor numbers were associated with less time spent grooming and sleeping/resting and more time spent playing and feeding/chewing.	[109]
Diana Monkey, ringtail lemur, cotton-top tamarin	Presence vs. absence	Behaviour	When visitors were present, aggression levels increased, and time spent engaged in grooming and other affiliative behaviours decreased.	[75]
	Height of visitors *	Behaviour	When visitors were asked to crouch, grooming behaviour increased and agonistic behaviour decreased.	
Golden-bellied mangabey	Number *	Behaviour	Animals moved to cages that were exposed to more visitors increased their aggressive displays towards visitors and increased within-group aggression. Animals displayed more threatening behaviour towards visitors than they did towards keepers or other primates.	[73]
Lion-tailed macaque	Presence vs. absence	Behaviour	Conducted at eight sites: On days when visitors were present, the frequencies of abnormal behavior, including self-biting, begging and bouncing, were significantly higher, and social behaviour and visibility were significantly lower.	[156]
Mandrill	Number	Behaviour	As visitor numbers increased, mandrills showed a reduction in time spent engaged in affiliative behaviour and increased time spent watching and threatening visitors.	[75]
Orangutan	Number	Behaviour	During periods of high visitor density, adults used paper sacks to cover their heads more and infants held onto adults more.	[77]
	Noise *	Behaviour	When confronted with noisy groups, animals spent more time looking at the visitors, and infants approached and held onto adults more.	
	Visual contact *	Behaviour	Orangutans showed a preference to position themselves facing towards the open window of the visitor viewing area.	[16]
	Number	Behaviour	A high visitor number increased time spent looking at visitors and begging.	[141]
	Behaviour	Behaviour	Visitors with food and visitors who were looking or taking photographs increased the time orangutans spent looking at visitors, begging and moving.	
	Proximity	Behaviour	Closer proximity between visitors and orangutans decreased the time orangutans spent playing/engaging in social behaviour and increased the time spent looking at visitors and begging.	
Pileated gibbon	Number	Behaviour	A higher visitor number was associated with increased levels of self-biting.	[94]
Ring-tailed and Mayotte lemurs, black spider monkey, white-fronted capuchin, Patas monkey, De Brazza’s monkey, Sykes monkey, talapoin, Barbary, lion-tailed and Sulawesi macaques and Hamadryas baboon	Number and behaviour	Behaviour	Animals showed more locomotion and directed more behavior towards visitors when confronted with small active and large active audiences rather than passive audiences.	[157]
Ring-tailed, mongoose and red-ruffed lemurs, squirrel monkey, Francois langur, spot-nosed monkey, De Brazza’s monkey, golden-bellied mangabey, gibbon, orangutan and chimpanzee	Number and behaviour	Behaviour	Animals directed more behaviour towards visitors when confronted with active audiences than passive audiences.	[79]
Ring-tailed lemur	Number and behaviour	Behaviour	As the number of visitors increased, time spent in locomotion and on the ground increased. However, visitor behaviour did not impact lemur behaviour.	[158]
	Number	Behaviour	Visitor numbers were associated with a decrease in foraging, resting and sunbathing and an increase in locomotion and alertness. However, these effects were reduced when weather was accounted for in the statistical model.	[72]
Siamang	Number	Behaviour	There was no difference detected in behaviour according to visitor number. However, siamangs appeared to respond to some human behaviours as they would to hostile behaviours from their own species.	[106]
Siamang, white-cheeked gibbon	Number	Behaviour	On days of higher visitor numbers, both siamangs and gibbons spent more time in areas away from the public and were less visible. There were no differences in rate of aggressive or affiliative interactions under different visitor numbers.	[85]
Sulawesi macaque	Number and noise	Behaviour	Conducted at five sites: As visitor numbers and noise increased, locomotion, vigilance and foraging increased and social huddling and resting decreased.	[159]
Western lowland gorilla	Visual contact *	Behaviour	Reducing visual contact with visitors resulted in lower levels of conspecific-directed aggression and stereotypies.	[12]
	Number and Noise	Behaviour and GC	High numbers of visitors and higher noise levels increased staring and charging at visitors and decreased food-related behaviour. No effects on GC concentration.	[61]
	Visual contact *	Behaviour and GC	When privacy screens were in place, staring and charging at visitors decreased. No effects on GC concentration.	
	Number	Behaviour	Conducted at two sites: One site found no effect of visitors and the other site found higher visitor numbers were associated with increase in duration of self-scratching and visual monitoring when no enrichment was provided.	[54]
	Number	Behaviour	Conducted on two groups: When large crowds were present, both groups were less visible. One group (bachelor group) also showed more aggressive behaviour with large crowds.	[92]
	Number	Behaviour	Conducted on four groups: Higher visitor numbers resulted in higher levels of stereotypies in two groups and males also showed increased aggression.	[131]
	Number	Behaviour (birth timing)	An analysis of the timing of 336 live gorilla births and 48 stillbirths at 53 accredited North American zoos from 1985–2014. Results showed no weekend (high visitor numbers) or weekday (low visitor numbers) effect on number of births or stillbirths.	[160]
	Number	Behaviour	There was no effect of crowd size on gorilla use of the areas of their exhibit closest to zoo visitors. In addition, they were observed in this area at a rate equal to or greater than expected by random movements at all three levels of crowd size analyzed.	[154]
	Number	Behaviour	High visitor numbers were associated with significantly more intragroup aggression, stereotypies and autogrooming, whereas a low visitor number was associated with a greater proportion of time spent resting.	[93]
White handed gibbon	Number and Noise	Behaviour	Higher numbers of visitors and higher noise levels resulted in increases in ‘look at public’ behaviour in all four gibbons. Higher noise levels also increased self-scratching behaviour in two individuals. One male showed an increase in aggressive ‘open mouth’ and ‘teeth display’ in response to the increased group size and noise level.	[55]
**Carnivora**				
Brown bear	Presence vs. absence	Behaviour	The presence of visitors was associated with greater levels of stereotypies, locomotion, vigilance and increased use of the back part of the enclosure.	[95]
Cheetah	Number and noise	Behaviour	No difference in cheetah behaviour was detected in response to visitor number.	[101]
Clouded leopard	Presence vs. absence	GCs	Higher GC concentrations in animals housed on display versus off display.	[40]
Eurasian lynx, ocelot, bobcat, jaguar, Asiatic lion	Presence vs. absence	Behaviour	Conducted at two sites: when visitors were present (zoo open), ocelots, lynx, bobcat and lions showed a decrease in activity and an increase in time spent further away from visitor areas, but the jaguar showed the opposite response.	[161]
Fennec fox	Number	Behaviour	Higher number of visitors was correlated with increased frequency of stereotypic running.	[35]
Giant panda	Presence vs. absence	Behaviour	Presence of visitors was associated with greater levels of exploration, feeding and time spent not visible. Pandas also showed an increase in use of the back part of the enclosure when visitors were present.	[95]
Harbour seal	Number	Behaviour	Under increasing visitor numbers, more seals were submerged under water.	[162]
Indian leopard	Presence vs. absence	Behaviour	Conducted at four sites: leopards rested significantly more when visitors were present.	[163]
Jaguar	Number and behaviour	Behaviour	When the visitor numbers and intensity of behaviour were lowest, jaguars spent more time non-visible. The female showed an increase in pacing behaviour at the intermediate level of intensity of visitor behaviour recorded.	[84]
	Presence vs. absence	Salivary cortisol	Conducted at two sites: At one site, ‘open to the public’ days were associated with increased levels of salivary cortisol compared to ‘no visitor’ days. There was no significant relationship detected at the other site.	[164]
Lion, Amur leopard, Amur tiger, Snow leopard, clouded leopard, fishing cat	Presence vs. absence	Behaviour	No effect of visitor presence or absence on felid activity patterns.	[15]
Puma	Number and noise	Behaviour	With higher numbers of visitors and noise levels, pumas increased time spent inactive and engaged in visitor-directed vigilance.	[98]
Meerkats	Visitor behaviour (noise) *	Behaviour	Conducted at three sites: No change in meerkat behaviour in response to a reduction in intensity of visitor behavior.	[14]
Mexican wolf	Number	Behaviour and GCs	Conducted at three sites: higher numbers of visitors were associated with higher GC concentration and less time spent lying and eating.	[97]
**Ungulates**				
Asian elephant, Indian rhino	Presence vs. absence	Salivary cortisol	Salivary cortisol concentrations were found to be significantly higher during the opening period (where animals had their first direct visual contact with visitors that year) compared to during pre- and post-opening periods.	[165]
Black rhino	Number	GCs	Higher mean GC concentrations were found at zoos where rhinos were maintained in enclosures that were exposed to the public around a greater portion of the perimeter.	[70]
Indian blackbuck	Number	Behaviour and GCs	Higher numbers of visitors were associated with higher GC concentration, increased levels of aggression and less time resting.	[100]
Indian gaur	Presence vs. absence	Behaviour	When visitors were present, animals showed higher levels of intragroup aggression and moving behavior and less resting behavior.	[90]
Sika deer	Number	Behaviour	High visitor numbers were associated with deer spending less time foraging and more time being watchful, resting and ‘non-visible’.	[87]
Soemmerring’s gazelle	Number	Behaviour	Conducted on three groups: animals in enclosures that were most accessible to visitors, had higher agonistic reactions than animals housed in enclosures with less exposure to visitors.	[88]
**Marsupials**				
Koala	Proximity	Behaviour	Greater numbers of visitors within a 5 m radius of koalas resulted in more visitor-vigilant behavior.	[78]
	Noise level *	Behaviour	When ‘loud’ crowd noise playbacks were played to koalas, they were significantly more likely to be disturbed than ‘quiet’ crowd noise playbacks.	
Quokka	Presence vs. absence *	Behaviour	Fewer quokkas were visible when the enclosure was open to visitors.	[76]
Red kangaroo and Kangaroo Island kangaroo	Visitor number	Behaviour and GCs	Conducted at two sites: when visitor numbers increased, both species of kangaroos increased time spent vigilant towards visitors and Kangaroo Island kangaroos increased time spent engaged in locomotion and decreased time spent resting. No effect of visitor numbers on faecal GC concentration or distance from path.	[86]
**Rodents**				
Black-tailed prairie dog	Number	Behaviour	Under higher visitor numbers, prairie dogs moved closer to visitors.	[108]
**Penguins**				
African penguin	Number	Behaviour	Presence of visitors in a pool adjacent to the penguin pool reduced the time penguins spent in their pool.	[99]
Gentoo penguin	Number	Behaviour	Higher numbers of visitors were associated with greater behavioural diversity and increased pool use by penguins. However, neither visitor behaviour nor enrichment appeared to affect behavioural diversity.	[110]
Little penguin	Presence vs. absence *	Behaviour	Presence of visitors increased levels of aggression, huddling and behaviours indicative of avoidance such as hiding and increased distance from viewing area.	[53]
**Other birds**				
Corella	Number	Behaviour	When there were fewer visitors present, Claude the corella spent more time engaging in ‘attention-seeking’ behaviours to initiate interaction with visitors.	[106]
Greater rhea	Presence vs. absence	Behaviour	In the presence of visitors, rheas increased walking alert behaviour.	[166]
